# 

**Published:** 1887-12-10

**Authors:** 


### CONTENTS.

**Not Alms, but a Friend** - - - - - - - 173

**Words of Consolation.**—Chapter LXI. The Sufferings of Christ - - - - - - - 174

**The Doctor's Pulpit.**—The Physic Bottle - - 175

**Common Accidents of Every-day Life.** By R. A. P. FORRESTER, B.A.—Bones, Fractures, Dislocations - 176

**Recollections of Hospital Life.** By an EX-PATIENT - 177

**The National Pension Fund.**—Opinions of the Press - 178

**The Matrons' Corner** - - - - - - 178

**Questions and Answers** - - - - - 178

**Professional Notes.** By GEO. W. POTTER, M.D. - - 179

**To Our Readers** - - - - - - 180

**Notes and News.**—Brompton Hospital for Consumption.—Vacancies.—Salford and Pendleton Royal Hospital.—Woolsorter's Disease.—The Sick, the Churches, and the Hospitals.—The Present *versus* the Stone Age.—The Manchester Hospital Saturday and Sunday Committees' Fund.—Madame Lind-Goldschmidt and the Worcester Infirmary.—Treatment of Consumption by Sulphur, etc. - - - - - 181

**Annotations.**—Mrs. Partington at the *Lancet* Office.—State-Supported Doctors - - - - - - 183

**Medicine Men of the World** By the MAN in the CROWD. Chapter V.—Savage and Civilised Practitioners - - - 184

**East and West.** By LESLIE KEITH. Chapter XI. - - 185

**Reminiscences of the Insane.** By D. HACK TUKE, M.D - 187

**The Editor's Letter-Box.**—Hospital Construction.—Nurses' Earnings.—Dreadnought Hospital - - - - - 188

**Amusements with Profit for all Ages.**—Competitions, Puzzles, Children's Corner, etc. - - - - - - iv

